# Predicting Chronic Wound Healing Time Using Machine Learning

**DOI:** 10.1089/wound.2021.0073

**Published:** 2022-03-24

**Authors:** Matthew Berezo, Joshua Budman, Daniel Deutscher, Cathy Thomas Hess, Kyle Smith, Deanna Hayes

**Affiliations:** Net Health Systems, Inc., Pittsburgh, Pennsylvania, USA.

**Keywords:** chronic wounds, informatics, personalized health care

## Abstract

**Objective:**

Chronic wounds have risen to epidemic proportions in the United States and can have an emotional, physical, and financial toll on patients. By leveraging data within the electronic health record (EHR), machine learning models offer the opportunity to facilitate earlier identification of wounds at risk of not healing or healing after an abnormally long time, which may improve treatment decisions and patient outcomes. Machine learning models in this study were built to predict chronic wound healing time.

**Approach:**

Machine learning models were developed using EHR data to predict patients at risk of having wounds not heal within 4, 8, and 12 weeks from the start of treatment. The models were trained on three data sets of 1,220,576 wounds, including 187 covariates describing patient demographics, comorbidities, and wound characteristics. The area under the receiver operating characteristic curve (AUC) was used to assess the accuracy of the models. Shapley Additive Explanations (SHAP) were used to analyze variable importance in predictions and enhance clinical interpretations.

**Results:**

The 4-, 8-, and 12-week gradient-boosted decision tree models achieved AUC's of 0.854, 0.855, and 0.853, respectively. Days in treatment, wound depth and location, and wound area were the most influential predictors of wounds at risk of not healing.

**Innovation:**

Machine learning models can accurately predict chronic wound healing time using EHR data. SHAP values can give insight into how patient-specific variables influenced predictions.

**Conclusion:**

Accurate models identifying patients with chronic wounds at risk of non or slow healing are feasible and can be incorporated into routine wound care.

**Figure:**
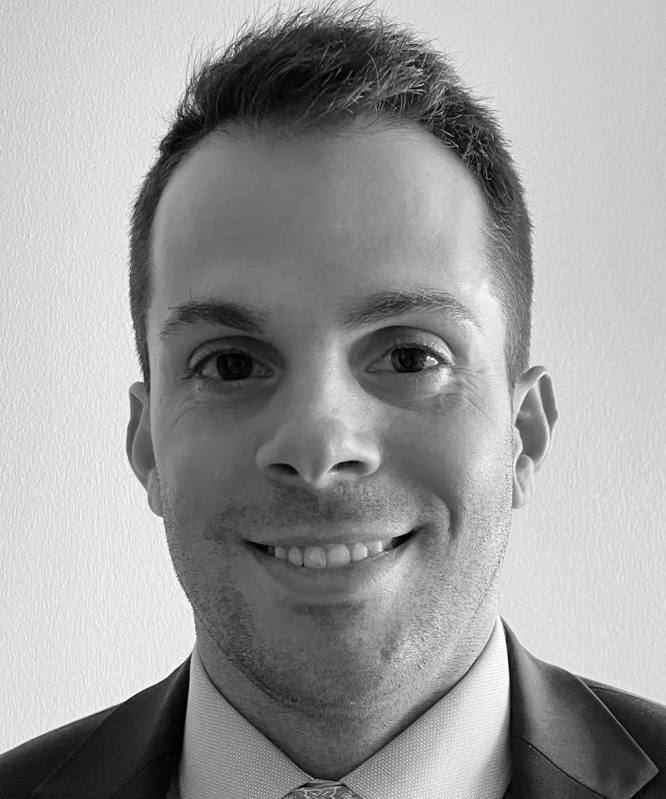
Matthew Berezo, BA

## INTRODUCTION

Chronic wounds, defined as not healing in a predictable or ordinary amount of time, with “ordinary” commonly defined as 4 to 12 weeks, affect an estimated 8.2 million Americans per year and can have an emotional, physical, and financial toll on patients.^1,2^ Chronic wounds are becoming more prevalent in the United States for multiple reasons, such as an aging population and increasing prevalence of obesity and diabetes.^3,4^ Without proper or timely treatment, patients with chronic wounds may face dire outcomes, including loss of limbs or even mortality. In addition to affecting a patient's quality of life, the cost of care for chronic wounds is substantial. Medicare costs for chronic wound treatments were estimated to be up to $96.8 billion in 2014, and the annual wound care product market is estimated to reach $18.7 billion by 2027.^2^

## CLINICAL PROBLEM ADDRESSED

Focus on early identification of patients at high risk of having wounds that will not heal or heal after an abnormal amount of time may enhance clinical decision making to limit complications, improve patient outcomes, and reduce costs of care. However, there are few predictive tools that allow clinicians to identify these high-risk individuals in a timely and accurate manner for any wound throughout the course of treatment while offering insight into factors that are affecting a patient's prognosis for healing.

Recent studies aimed to predict chronic wound healing time using machine learning.^3,5,6^ However, most of these studies only made predictions at specific times during treatment, focused on a limited number of wound types, had lower accuracy, or did not offer patient-specific insight into variable importance in predicting wound healing in terms of weight and whether the influence tended to be positive or negative.

Cho *et al.* reported models that predict chronic would healing time.^3^ However, the models only provided predictions upon patients' baseline presentation and focused on the 12-week healing timeframe, yielding and area under the receiver operating characteristic curve (AUC) of 0.71.^3^ Fife *et al.* developed a machine learning model to predict diabetic foot ulcer chronic wound healing time, achieving AUC levels of 0.67.^5^ Jung *et al.* developed machine learning models to predict wound healing in the 15-week timeframe, achieving an AUC of 0.84.^6^ While achieving higher accuracy, this study also focused on predictions at one time point and did not make predictions for subsequent visits.

Additionally, the existing models described above evaluated predictive importance in ways that do not capture local interpretations, that is, patient-specific characteristics that are unique driving factors for a patient's risk of not healing. For example, variable importance analyses for algorithms such as linear or logistic regression is typically done by analyzing coefficient values that are static and generalize for the population. In tree-based methods such as decision trees and random forests, no interpretation of directional is typically offered, and only global importance of the variable is presented. These types of variable importance analyses were used in the studies mentioned above.

Accordingly, this study's goal was to build highly accurate machine learning models that can be used in real-time to predict the probability of chronic wounds not healing in 4, 8, and 12 weeks from the start of treatment across multiple time points. The analysis also gives insight into variable importance in terms of magnitude and directionality at the global and local levels as patient and wound characteristics change over the course of treatment. To the best of our knowledge, this has not been previously explored in wound care research.

## MATERIALS AND METHODS

### Population derivation

Retrospective data were sourced from Net Health Systems, Inc.'s Wound Care Analysis Data Set. The study was deemed exempt from informed consent requirements after review by IRB Solutions, a private Institutional Review Board located in Yarnell, Arizona. These data represent patients with chronic wounds from 595 facilities across 47 states in the United States between January 1, 2012 and July 1, 2021. The models included 187 independent variables describing patient demographics, comorbidities, time in days from the start of treatment, and wound characteristics such as type and status from the previous and current visits. Independent variables were chosen with clinical guidance and review of published literature.^3–12^ Supplementary Data S1 contains the list of variables that were used in the analysis.

To protect the model from being negatively impacted by outliers, we removed all records for patients who had implausible values for wound surface area, length, width, and depth.^6^ These outliers were determined by manually observing the distribution of these values across the population. This process identified 12,029 wounds whose treatment visits were dropped from the analysis.

A total of 461,293 patients were included in the analysis, accounting for 1,220,576 wounds. Of these wounds, 542,723 (45%) healed within 4 weeks from the start of treatment, 789,775 (65%) healed within 8 weeks from the start of treatment, and 909,113 (75%) healed within 12 weeks from the start of treatment. Table 1 displays clinical summary statistics for these patients.^13^

**Table 1.** Chronic wound patients grouped by healing

Grouped by Healing	Missing	Overall	Healed	Not Healed
*N*		461,293	416,804	44,489
Wound type, *n* (%)	0			
Arterial ulcer		7,715 (1.7)	6,348 (1.5)	1,367 (3.1)
Burn		11,238 (2.4)	10,395 (2.5)	843 (1.9)
Cellulitis		7,598 (1.6)	6,980 (1.7)	618 (1.4)
Diabetic ulcer		60,919 (13.2)	52,318 (12.6)	8,601 (19.3)
Other		80,525 (17.5)	73,374 (17.6)	7,151 (16.1)
Pressure ulcer		74,371 (16.1)	66,401 (15.9)	7,818 (17.6)
Skin tear		11,933 (2.6)	11,594 (2.8)	339 (0.8)
Surgical wound		72,074 (15.6)	64,256 (15.4)	7,818 (17.6)
Trauma wound		59,784 (13.0)	55,288 (13.3)	4,496 (10.1)
Venous ulcer		75,136 (16.3)	69,850 (16.8)	5,286 (11.9)
Body part, *n* (%)	0			
Foot		47,704 (10.3)	41,946 (10.1)	5,758 (12.9)
Lower leg		121,179 (26.3)	112,511 (27.0)	8,668 (19.5)
Other		242,083 (52.5)	218,608 (52.4)	23,475 (52.8)
Toe		45,602 (9.9)	39,399 (9.5)	6,203 (13.9)
Upper leg		4,725 (1.0)	4,340 (1.0)	385 (0.9)
Initial wound area (mm^2^), mean (SD)	0			
		1,135.4 (4,772.8)	1,127.2 (4,890.8)	1,211.5 (3,482.1)
Age, mean (SD)	632			
		65.7 (21.8)	66.2 (20.0)	61.2 (33.6)
Concurrent wounds, mean (SD)	0			
		1.8 (1.3)	1.8 (1.3)	1.7 (1.2)
Gender	7,607			
Female		223,859 (49.3)	203,648 (49.7)	20,211 (45.9)
Male		229,827 (50.7)	206,019 (50.3)	23,808 (54.1)
Stage depth, *n* (%)	0			
Eschar covered		11,508 (2.5)	10,107 (2.4)	1,401 (3.1)
Full thickness		199,294 (43.2)	180,692 (43.4)	18,602 (41.8)
Grade 0		1,237 (0.3)	1,154 (0.3)	83 (0.2)
Grade 1		19,957 (4.3)	17,785 (4.3)	2,172 (4.9)
Grade 2		14,983 (3.2)	12,872 (3.1)	2,111 (4.7)
Grade 3		7,994 (1.7)	6,467 (1.6)	1,527 (3.4)
Grade 4		2,063 (0.4)	1,483 (0.4)	580 (1.3)
Grade 5		84 (0.0)	63 (0.0)	21 (0.0)
Partial thickness		62,575 (13.6)	58,935 (14.1)	3,640 (8.2)
Stage 1 Pressure injury		2,677 (0.6)	2,603 (0.6)	74 (0.2)
Stage 2 Pressure injury		20,728 (4.5)	19,341 (4.6)	1,387 (3.1)
Stage 3 Pressure injury		25,203 (5.5)	22,856 (5.5)	2,347 (5.3)
Stage 4 Pressure injury		5,719 (1.2)	4,245 (1.0)	1,474 (3.3)
Unknown		87,271 (18.9)	78,201 (18.8)	9,070 (20.4)
Eschar, *n* (%)	421,537			
1–25%		9,703 (24.4)	8,270 (24.0)	1,433 (26.8)
26–50%		4,449 (11.2)	3,689 (10.7)	760 (14.2)
51–75%		3,464 (8.7)	2,833 (8.2)	631 (11.8)
76–100%		18,938 (47.6)	16,523 (48.0)	2,415 (45.2)
None		644 (1.6)	608 (1.8)	36 (0.7)
Yes		2,558 (6.4)	2,492 (7.2)	66 (1.2)
Current infection, *n* (%)	0			
No		433,722 (94.0)	393,742 (94.5)	39,980 (89.9)
Yes		27,571 (6.0)	23,062 (5.5)	4,509 (10.1)
History of tobacco use, *n* (%)	0			
No		461,077 (100.0)	416,619 (100.0)	44,458 (99.9)
Yes		216 (0.0)	185 (0.0)	31 (0.1)
Dementia/Alzheimer's, *n* (%)	0			
No		447,921 (97.1)	404,995 (97.2)	42,926 (96.5)
Yes		13,372 (2.9)	11,809 (2.8)	1,563 (3.5)
Coronary artery disease, *n* (%)	0			
No		423,835 (91.9)	383,381 (92.0)	40,454 (90.9)
Yes		37,458 (8.1)	33,423 (8.0)	4,035 (9.1)
Congestive heart failure	0			
No		425,163 (92.2)	384,633 (92.3)	40,530 (91.1)
Yes		36,130 (7.8)	32,171 (7.7)	3,959 (8.9)
Chronic obstructive pulmonary disorder, *n* (%)	0			
No		424,498 (92.0)	383,877 (92.1)	40,621 (91.3)
Yes		36,795 (8.0)	32,927 (7.9)	3,868 (8.7)
Peripheral vascular disease, *n* (%)	0			
No		441,372 (95.7)	399,369 (95.8)	42,003 (94.4)
Yes		19,921 (4.3)	17,435 (4.2)	2,486 (5.6)
Paraplegia, *n* (%)	0			
No		457,951 (99.3)	413,971 (99.3)	43,980 (98.9)
Yes		3,342 (0.7)	2,833 (0.7)	509 (1.1)
Hypertension, *n* (%)	0			
No		253,508 (55.0)	229,460 (55.1)	24,048 (54.1)
Yes		207,785 (45.0)	187,344 (44.9)	20,441 (45.9)

SD, standard deviation.

Before model training, patients were randomly split into training (60% unique patients), validation (15%), and testing (25%) sets, which ensured that a patient was only represented in one of the three populations. This strategy was used to help prevent overfitting during model development. Wound treatment visits for these patients were then used during model creation, where the algorithms learned on the training and validation sets were tested on the held-out testing set. The training, validation, and testing populations for each model that were also censored to ensure visits that occurred after the healing timeframe of interest were not included in model development. For example, it is common for a patient to remain in treatment for longer than 4, 8, or 12 weeks. Treatments that occurred after the target timeframe were not included in the analysis. Additionally, final treatment visits, where the days in treatment was equal to the total days in treatment, were dropped from the analysis to ensure that the model was developed on observations before an outcome of healed or not healed. Therefore, the data sets for each model had different numbers of observations. The 4-, 8-, and 12-week models were trained and tested on 3,477,501, 4,580,575, and 5,210,023 treatment visits, respectively. Tables 2–4 display clinical summary statistics for wounds in each population at patient presentation, stratified by those that did and did not heal within 4, 8, and 12 weeks.^13^ Wounds were defined as “healed” if they had a final wound status labeled as “Resolved,” “Healed,” “Graft,” “Closed,” or “Treatment Complete” in the electronic health record (EHR) before the timeframe of interest. Nonhealing wounds were defined as those with a final wound status label of “Not Healed,” “Amputated,” “Quit Treatment,” or healing after the timeframe of interest. These labels were chosen with clinical guidance and review of published literature.^3,6,14^

**Table 2.** Chronic wounds grouped by healing in 4 weeks

Grouped by Healing in 4 Weeks	Missing	Overall	Healed	Not Healed
*N*		1,220,576	542,723	677,853
Wound type, *n* (%)	0			
Arterial ulcer		22,547 (1.8)	6,048 (1.1)	16,499 (2.4)
Burn		24,438 (2.0)	13,928 (2.6)	10,510 (1.6)
Cellulitis		15,531 (1.3)	8,081 (1.5)	7,450 (1.1)
Diabetic ulcer		179,396 (14.7)	58,987 (10.9)	120,409 (17.8)
Other		214,282 (17.6)	109,578 (20.2)	104,704 (15.4)
Pressure ulcer		185,537 (15.2)	76,100 (14.0)	109,437 (16.1)
Skin tear		43,748 (3.6)	31,851 (5.9)	11,897 (1.8)
Surgical wound		126,963 (10.4)	41,963 (7.7)	85,000 (12.5)
Trauma wound		149,705 (12.3)	77,186 (14.2)	72,519 (10.7)
Venous ulcer		258,429 (21.2)	119,001 (21.9)	139,428 (20.6)
Body part, *n* (%)	0			
Foot		131,952 (10.8)	45,142 (8.3)	86,810 (12.8)
Lower leg		358,998 (29.4)	169,288 (31.2)	189,710 (28.0)
Other		569,455 (46.7)	262,214 (48.3)	307,241 (45.3)
Toe		147,038 (12.0)	59,553 (11.0)	87,485 (12.9)
Upper leg		13,133 (1.1)	6,526 (1.2)	6,607 (1.0)
Initial wound area (mm^2^), mean (SD)	0			
		1,022.7 (4,806.7)	727.7 (3,491.6)	1,260.7 (5,637.7)
Age, mean (SD)	1,116			
		66.2 (21.2)	67.7 (19.2)	65.1 (22.6)
Concurrent wounds, mean (SD)	0			
		2.8 (2.2)	3.0 (2.3)	2.6 (2.0)
Gender	15,918			
Female		539,960 (44.8)	233,386 (43.7)	306,574 (45.7)
Male		664,698 (55.2)	300,363 (56.3)	364,335 (54.3)
Stage depth, *n* (%)	0			
Eschar covered		32,280 (2.6)	16,054 (3.0)	16,226 (2.4)
Full thickness		473,371 (38.8)	186,716 (34.4)	286,655 (42.3)
Grade 0		4,778 (0.4)	2,577 (0.5)	2,201 (0.3)
Grade 1		66,348 (5.4)	26,753 (4.9)	39,595 (5.8)
Grade 2		40,844 (3.3)	11,225 (2.1)	29,619 (4.4)
Grade 3		17,883 (1.5)	2,432 (0.4)	15,451 (2.3)
Grade 4		5,336 (0.4)	689 (0.1)	4,647 (0.7)
Grade 5		199 (0.0)	27 (0.0)	172 (0.0)
Partial thickness		206,516 (16.9)	129,455 (23.9)	77,061 (11.4)
Stage 1 Pressure injury		7,805 (0.6)	5,218 (1.0)	2,587 (0.4)
Stage 2 Pressure injury		56,142 (4.6)	30,611 (5.6)	25,531 (3.8)
Stage 3 Pressure injury		57,370 (4.7)	19,066 (3.5)	38,304 (5.7)
Stage 4 Pressure injury		11,334 (0.9)	895 (0.2)	10,439 (1.5)
Unknown		240,370 (19.7)	111,005 (20.5)	129,365 (19.1)
Eschar, *n* (%)	1,109,752			
1–25%		23,721 (21.4)	8,385 (19.1)	15,336 (22.9)
26–50%		11,754 (10.6)	4,158 (9.5)	7,596 (11.3)
51–75%		9,111 (8.2)	3,194 (7.3)	5,917 (8.8)
76–100%		56,859 (51.3)	24,904 (56.8)	31,955 (47.7)
None		1,475 (1.3)	665 (1.5)	810 (1.2)
Yes		7,904 (7.1)	2,549 (5.8)	5,355 (8.0)
Current infection, *n* (%)	0			
No		1,157,395 (94.8)	518,130 (95.5)	639,265 (94.3)
Yes		63,181 (5.2)	24,593 (4.5)	38,588 (5.7)
History of tobacco use, *n* (%)	0			
No		1,220,046 (100.0)	542,511 (100.0)	677,535 (100.0)
Yes		530 (0.0)	212 (0.0)	318 (0.0)
Dementia/Alzheimer's, *n* (%)	0			
No		1,186,725 (97.2)	526,457 (97.0)	660,268 (97.4)
Yes		33,851 (2.8)	16,266 (3.0)	17,585 (2.6)
Coronary artery disease, *n* (%)	0			
No		1,103,918 (90.4)	490,593 (90.4)	613,325 (90.5)
Yes		116,658 (9.6)	52,130 (9.6)	64,528 (9.5)
Congestive heart failure	0			
No		1,100,034 (90.1)	487,558 (89.8)	612,476 (90.4)
Yes		120,542 (9.9)	55,165 (10.2)	65,377 (9.6)
Chronic obstructive pulmonary disorder, *n* (%)	0			
No		1,111,453 (91.1)	492,713 (90.8)	618,740 (91.3)
Yes		109,123 (8.9)	50,010 (9.2)	59,113 (8.7)
Peripheral vascular disease, *n* (%)	0			
No		1,147,372 (94.0)	513,885 (94.7)	633,487 (93.5)
Yes		73,204 (6.0)	28,838 (5.3)	44,366 (6.5)
Paraplegia, *n* (%)	0			
No		1,206,577 (98.9)	538,321 (99.2)	668,256 (98.6)
Yes		13,999 (1.1)	4,402 (0.8)	9,597 (1.4)
Hypertension, *n* (%)	0			
No		630,941 (51.7)	284,703 (52.5)	346,238 (51.1)
Yes		589,635 (48.3)	258,020 (47.5)	331,615 (48.9)

**Table 3.** Chronic wounds grouped by healing in 8 weeks

Grouped by Healing in 8 Weeks	Missing	Overall	Healed	Not Healed
*N*		1,220,576	789,775	430,801
Wound type, *n* (%)	0			
Arterial ulcer		22,547 (1.8)	10,132 (1.3)	12,415 (2.9)
Burn		24,438 (2.0)	18,947 (2.4)	5,491 (1.3)
Cellulitis		15,531 (1.3)	11,267 (1.4)	4,264 (1.0)
Diabetic ulcer		179,396 (14.7)	94,063 (11.9)	85,333 (19.8)
Other		214,282 (17.6)	150,225 (19.0)	64,057 (14.9)
Pressure ulcer		185,537 (15.2)	110,811 (14.0)	74,726 (17.3)
Skin tear		43,748 (3.6)	38,894 (4.9)	4,854 (1.1)
Surgical wound		126,963 (10.4)	72,758 (9.2)	54,205 (12.6)
Trauma wound		149,705 (12.3)	110,393 (14.0)	39,312 (9.1)
Venous ulcer		258,429 (21.2)	172,285 (21.8)	86,144 (20.0)
Body part, *n* (%)	0			
Foot		131,952 (10.8)	71,217 (9.0)	60,735 (14.1)
Lower leg		358,998 (29.4)	246,074 (31.2)	112,924 (26.2)
Other		569,455 (46.7)	373,706 (47.3)	195,749 (45.4)
Toe		147,038 (12.0)	89,437 (11.3)	57,601 (13.4)
Upper leg		13,133 (1.1)	9,341 (1.2)	3,792 (0.9)
Initial wound area (mm^2^), mean (SD)		1,022.7 (4,806.7)	843.3 (4,391.0)	1,353.6 (5,476.0)
				
Age, mean (SD)	1,116			
		66.2 (21.2)	67.2 (18.4)	64.5 (25.5)
Concurrent wounds, mean (SD)	0			
		2.8 (2.2)	2.9 (2.2)	2.6 (2.0)
Gender	15,918			
Female		539,960 (44.8)	346,891 (44.6)	193,069 (45.2)
Male		664,698 (55.2)	430,980 (55.4)	233,718 (54.8)
Stage depth, *n* (%)	0			
Eschar covered		32,280 (2.6)	22,270 (2.8)	10,010 (2.3)
Full thickness		473,371 (38.8)	294,913 (37.3)	178,458 (41.4)
Grade 0		4,778 (0.4)	3,589 (0.5)	1,189 (0.3)
Grade 1		66,348 (5.4)	40,578 (5.1)	25,770 (6.0)
Grade 2		40,844 (3.3)	19,304 (2.4)	21,540 (5.0)
Grade 3		17,883 (1.5)	5,336 (0.7)	12,547 (2.9)
Grade 4		5,336 (0.4)	1,398 (0.2)	3,938 (0.9)
Grade 5		199 (0.0)	44 (0.0)	155 (0.0)
Partial thickness		206,516 (16.9)	166,032 (21.0)	40,484 (9.4)
Stage 1 Pressure injury		7,805 (0.6)	6,484 (0.8)	1,321 (0.3)
Stage 2 Pressure injury		56,142 (4.6)	40,922 (5.2)	15,220 (3.5)
Stage 3 Pressure injury		57,370 (4.7)	31,098 (3.9)	26,272 (6.1)
Stage 4 Pressure injury		11,334 (0.9)	1,999 (0.3)	9,335 (2.2)
Unknown		240,370 (19.7)	155,808 (19.7)	84,562 (19.6)
Eschar, *n* (%)	1,109,752			
1–25%		23,721 (21.4)	13,085 (19.9)	10,636 (23.6)
26–50%		11,754 (10.6)	6,393 (9.7)	5,361 (11.9)
51–75%		9,111 (8.2)	4,892 (7.4)	4,219 (9.4)
76–100%		56,859 (51.3)	36,238 (55.1)	20,621 (45.7)
None		1,475 (1.3)	955 (1.5)	520 (1.2)
Yes		7,904 (7.1)	4,172 (6.3)	3,732 (8.3)
Current infection, *n* (%)	0			
No		1,157,395 (94.8)	753,061 (95.4)	404,334 (93.9)
Yes		63,181 (5.2)	36,714 (4.6)	26,467 (6.1)
History of tobacco use, *n* (%)	0			
No		1,220,046 (100.0)	789,463 (100.0)	430,583 (99.9)
Yes		530 (0.0)	312 (0.0)	218 (0.1)
Dementia/Alzheimer's, *n* (%)	0			
No		1,186,725 (97.2)	767,249 (97.1)	419,476 (97.4)
Yes		33,851 (2.8)	22,526 (2.9)	11,325 (2.6)
Coronary artery disease, *n* (%)	0			
No		1,103,918 (90.4)	714,254 (90.4)	389,664 (90.5)
Yes		116,658 (9.6)	75,521 (9.6)	41,137 (9.5)
Congestive heart failure	0			
No		1,100,034 (90.1)	710,294 (89.9)	389,740 (90.5)
Yes		120,542 (9.9)	79,481 (10.1)	41,061 (9.5)
Chronic obstructive pulmonary disorder, *n* (%)	0			
No		1,111,453 (91.1)	717,288 (90.8)	394,165 (91.5)
Yes		109,123 (8.9)	72,487 (9.2)	36,636 (8.5)
Peripheral vascular disease, *n* (%)	0			
No		1,147,372 (94.0)	746,126 (94.5)	401,246 (93.1)
Yes		73,204 (6.0)	43,649 (5.5)	29,555 (6.9)
Paraplegia, *n* (%)	0			
No		1,206,577 (98.9)	782,772 (99.1)	423,805 (98.4)
Yes		13,999 (1.1)	7,003 (0.9)	6,996 (1.6)
Hypertension, *n* (%)	0			
No		630,941 (51.7)	411,073 (52.0)	219,868 (51.0)
Yes		589,635 (48.3)	378,702 (48.0)	210,933 (49.0)

**Table 4.** Chronic wounds grouped by healing in 12 weeks

Grouped by Healing in 12 Weeks	Missing	Overall	Healed	Not Healed
*N*		1,220,576	909,113	311,463
Wound type, *n* (%)	0			
Arterial ulcer		22,547 (1.8)	12,705 (1.4)	9,842 (3.2)
Burn		24,438 (2.0)	20,835 (2.3)	3,603 (1.2)
Cellulitis		15,531 (1.3)	12,578 (1.4)	2,953 (0.9)
Diabetic ulcer		179,396 (14.7)	113,834 (12.5)	65,562 (21.0)
Other		214,282 (17.6)	168,452 (18.5)	45,830 (14.7)
Pressure ulcer		185,537 (15.2)	128,583 (14.1)	56,954 (18.3)
Skin tear		43,748 (3.6)	40,854 (4.5)	2,894 (0.9)
Surgical wound		126,963 (10.4)	89,226 (9.8)	37,737 (12.1)
Trauma wound		124,301 (13.7)	124,301 (13.7)	25,404 (8.2)
Venous ulcer		258,429 (21.2)	197,745 (21.8)	60,684 (19.5)
Body part, *n* (%)	0			
Foot		131,952 (10.8)	86,016 (9.5)	45,936 (14.7)
Lower leg		358,998 (29.4)	281,878 (31.0)	77,120 (24.8)
Other		569,455 (46.7)	426,216 (46.9)	143,239 (46.0)
Toe		147,038 (12.0)	104,420 (11.5)	42,618 (13.7)
Upper leg		13,133 (1.1)	10,583 (1.2)	2,550 (0.8)
Initial wound area (mm^2^), mean (SD)	0			
		1,022.7 (4,806.7)	899.9 (4,601.7)	1,383.0 (5,346.9)
Age, mean (SD)	1,116			
		66.2 (21.2)	67.0 (18.6)	64.1 (27.3)
Concurrent wounds, mean (SD)	0			
		2.8 (2.2)	2.9 (2.2)	2.5 (2.0)
Gender	15,918			
Female		539,960 (44.8)	402,111 (44.9)	137,849 (44.6)
Male		664,698 (55.2)	493,793 (55.1)	170,905 (55.4)
Stage depth, *n* (%)	0			
Eschar covered		32,280 (2.6)	24,862 (2.7)	7,418 (2.4)
Full thickness		473,371 (38.8)	349,381 (38.4)	123,990 (39.8)
Grade 0		4,778 (0.4)	3,931 (0.4)	847 (0.3)
Grade 1		66,348 (5.4)	47,550 (5.2)	18,798 (6.0)
Grade 2		40,844 (3.3)	24,255 (2.7)	16,589 (5.3)
Grade 3		17,883 (1.5)	7,717 (0.8)	10,166 (3.3)
Grade 4		5,336 (0.4)	1,997 (0.2)	3,339 (1.1)
Grade 5		199 (0.0)	70 (0.0)	129 (0.0)
Partial thickness		206,516 (16.9)	179,200 (19.7)	27,316 (8.8)
Stage 1 Pressure injury		7,805 (0.6)	6,918 (0.8)	887 (0.3)
Stage 2 Pressure injury		56,142 (4.6)	45,452 (5.0)	10,690 (3.4)
Stage 3 Pressure injury		57,370 (4.7)	37,850 (4.2)	19,520 (6.3)
Stage 4 Pressure injury		11,334 (0.9)	3,087 (0.3)	8,247 (2.6)
Unknown		240,370 (19.7)	176,843 (19.5)	63,527 (20.4)
Eschar, *n* (%)	1,109,752			
1–25%		23,721 (21.4)	15,492 (20.2)	8,229 (24.0)
26–50%		11,754 (10.6)	7,574 (9.9)	4,180 (12.2)
51–75%		9,111 (8.2)	5,772 (7.5)	3,339 (9.8)
76–100%		56,859 (51.3)	41,325 (53.9)	15,534 (45.4)
None		1,475 (1.3)	1,133 (1.5)	342 (1.0)
Yes		7,904 (7.1)	5,303 (6.9)	2,601 (7.6)
Current infection, *n* (%)	0			
No		1,157,395 (94.8)	866,511 (95.3)	290,884 (93.4)
Yes		63,181 (5.2)	42,602 (4.7)	20,579 (6.6)
History of tobacco use, *n* (%)	0			
No		1,220,046 (100.0)	908,738 (100.0)	311,308 (100.0)
Yes		530 (0.0)	375 (0.0)	155 (0.0)
Dementia/Alzheimer's, *n* (%)	0			
No		1,186,725 (97.2)	883,714 (97.2)	303,011 (97.3)
Yes		33,851 (2.8)	25,399 (2.8)	8,452 (2.7)
Coronary artery disease, *n* (%)	0			
No		1,103,918 (90.4)	822,350 (90.5)	281,568 (90.4)
Yes		116,658 (9.6)	86,763 (9.5)	29,895 (9.6)
Congestive heart failure	0			
No		1,100,034 (90.1)	818,251 (90.0)	281,783 (90.5)
Yes		120,542 (9.9)	90,862 (10.0)	29,680 (9.5)
Chronic obstructive pulmonary disorder, *n* (%)	0			
No		1,111,453 (91.1)	826,356 (90.9)	285,097 (91.5)
Yes		109,123 (8.9)	82,757 (9.1)	26,366 (8.5)
Peripheral vascular disease, *n* (%)	0			
No		1,147,372 (94.0)	858,070 (94.4)	289,302 (92.9)
Yes		73,204 (6.0)	51,043 (5.6)	22,161 (7.1)
Paraplegia, *n* (%)	0			
No		1,206,577 (98.9)	900,718 (99.1)	305,859 (98.2)
Yes		13,999 (1.1)	8,395 (0.9)	5,604 (1.8)
Hypertension, *n* (%)	0			
No		630,941 (51.7)	471,699 (51.9)	159,242 (51.1)
Yes		589,635 (48.3)	437,414 (48.1)	152,221 (48.9)

### Model development

All models were developed in Python 3.8.5. Logistic regression, random forests, gradient-boosted decision trees (GBDT), and deep feedforward neural networks (DNN) were the tested machine learning methods to predict chronic wound healing probability in each timeframe. We applied different data preparation and feature engineering based on each algorithm's mechanism of learning.

For all models, we coded missing data in nominal categorical variables as a separate category, “Missing.” We used one-hot encoding for the logistic regression, random forest, and deep feedforward neural network models. We used integer-encoding for the GBDT models.^15,16^

For the logistic regression and DNN models, we used the mean value by wound type to impute missing data in continuous variables. After imputation, we normalized these continuous variables and scaled them using z-scores. For the random forest models, we did not normalize or scale the continuous variables since the algorithm makes split decisions instead of deriving weighted parameters and are therefore not affected by scaling or normalization. Furthermore, for the GBDT models, we did not use imputation for missing values of continuous variables because GBDT handle missing data without imputation and makes split decisions instead of deriving weighted parameters, like random forests.

The logistic regression and random forest models were developed using scikit-learn.^17^ Least absolute shrinkage and selection operator was used for model regularization and variable selection in the logistic regression models.

For random forest models, number of trees, maximum depth, minimum samples per split and per leaf, and maximum features were the hyperparameters tuned during model development. The functionalities of these hyperparameters are described in Supplemental Data S2.

LightGBM 3.0.0, a GBDT package developed by Microsoft, was used for the GBDT models. GBDT's are typically top performers for structured, tabular regression, and classification problems.^18^ For the GBDT models, learning rate, evaluation metric, maximum depth, early stopping rounds, column sample by tree, number of leaves, L1 regularization, L2 regularization, and maximum bins were the hyperparameters tuned during model development. The functionalities of these hyperparameters are also described in Supplemental Data S2.

Initial hyperparameters were chosen using scikit-hyperband. Hyperband iteratively fits models with different combinations of hyperparameter values and evaluate results concerning the target metric, using a bandit-based, successive halving approach to choose final parameters.^19^ For these models, hyperband chose parameters in the regions of expected highest AUC for each trial. After tuning with hyperband, hyperparameters were again tuned manually to avoid overfitting. Once final hyperparameters were chosen, the algorithms were given 100,000 epochs to learn during training while optimizing for binary logarithmic loss.

The DNN models were developed in Keras using the sequential framework.^18^ A suitable model architecture was chosen for the problem: several initial dense layers with a rectified linear unit activation function followed by a dropout layer to combat overfitting. The final dense layer had a sigmoid activation function to match the binary target space.

We evaluated the algorithm results with AUC on the training and testing sets for each model. Furthermore, we analyzed each models' ability to discriminate with confusion matrices on their testing sets to evaluate accuracy, sensitivity, specificity, positive predictive value (PPV), negative predictive value (NPV), and F1-score. The confusion matrices' discrimination thresholds were chosen using Youden's J-statistic, a Pareto-optimal value to optimize sensitivity and specificity.

### Model interpretability

For clinical practice, it is important to evaluate how variables contribute to a prediction in terms of magnitude of importance and directionality. We used Shapley Additive Explanations (SHAP) to assess model interpretability. SHAP provides information on variable importance for models that have traditionally been difficult to interpret. SHAP values are derived by considering all possible permutations of independent variables for a single prediction and then assigning marginal contributions, or SHAP values, to each variable in the prediction.^20^ In our models, a positive SHAP value (>0) indicates that the variable influenced a higher probability that the wound would heal in the target timeframe in the context of a single observation (*e.g.*, treatment visit), whereas a negative SHAP value indicates the opposite. The absolute value of the SHAP value indicates which variables had more weight, or importance, in a prediction. This allows for a rich interpretation of the magnitude and direction in which a variable affected a prediction for a specific patient and wound. The ability to find unique, patient-level insights of predictions is what differentiates SHAP from other traditional variable importance analyses. SHAP values for a variable may differ from one patient to another because of their unique combination of covariates and interactions with one another. This is unlike parameterized models such as linear and logistic regression, which produce coefficients that are static and applicable on a population level, or nonparameterized models like decision trees and random forests in which historically popular measures such as Gini importance, gain, or entropy also represent global importance but provide no directionality. When SHAP values for each variable are compared across many predictions, SHAP also allows for a global interpretation of how covariates affected predictions across the population. Examples from the results of this study are provided below.

## RESULTS

**Figure 1.**
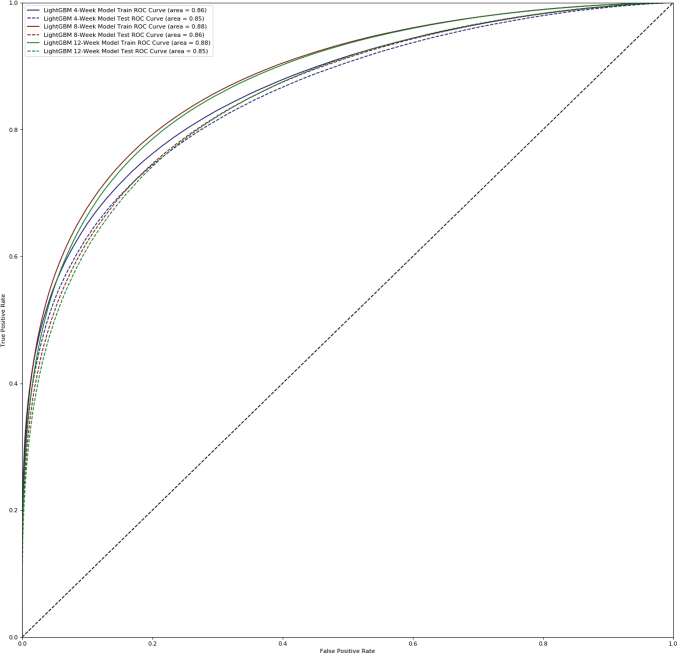
Comparison of ROC curves and AUC for the top performing GBDT models for the 4-, 8-, and 12-week models for both training and testing sets. The training and test AUC's for each model are similar and do not suggest overfitting. *Blue:* 4-week training set ROC curve. *Blue dashed*: 4-week testing set ROC curve. *Red*: 8-week training set ROC curve. *Red dashed*: 8-week testing set ROC curve. *Green*: 12-week training set ROC curve. *Green Dashed*: 12-week testing set ROC curve. AUC, area under the ROC curve; GBDT, gradient-boosted decision trees; ROC, receiver operating characteristic.

The GBDT models substantially outperformed the logistic regression, random forest, and DNN models across all target timepoints. Table 5 displays testing set AUC's for each model and target timeframe. Figure 1 shows the receiver operating characteristic (ROC) curves for the GBDT models on the training and testing populations. The GBDT models predicting wound healing in 4, 8, and 12 weeks from the start of treatment achieved AUC's of 0.854, 0.855, and 0.853 on their testing sets, respectively. The training set and testing set AUC's are similar and do not suggest overfitting.

**Table 5.** Model comparisons by testing set area under the receiver operating characteristic curve's

Model Type	4-Week AUC	8-Week AUC	12-Week AUC
Logistic regression	0.792	0.814	0.815
Random forest	0.818	0.818	0.817
Neural network	0.795	0.805	0.788
LigthGBM GBDT	0.854	0.855	0.853

AUC, area under the receiver operating characteristic curve; GBDT, gradient-boosted decision trees.

Table 6 shows the sensitivity, specificity, PPV, NPV, and F1-score metrics for the confusion matrices derived on the testing sets for the 4-, 8-, and 12-week GBDT models.

**Table 6.** Testing set confusion matrix statistics for the top-performing gradient-boosted decision trees models for the 4-, 8-, and 12-week target timeframes

Target Timeframe	Accuracy (%)	Sensitivity (%)	Specificity (%)	PPV (%)	NPV (%)	F1-Score
4-Week	77	77	7	59	88	0.671
8-Week	77	77	77	75	79	0.759
12-Week	79	77	77	82	71	0.793

NPV, negative predictive value; PPV, positive predictive value.

**Figure 2.**
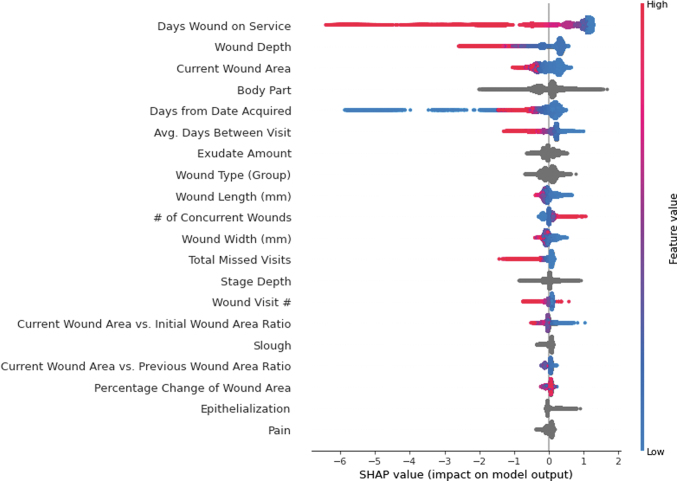
SHAP summary plot for the top 20 most influential variables in the 4-week GBDT predictive model. Variables are listed in descending order of overall influence in the model on the *left-hand side*. Parameter values for variables of individual observations are plotted to the *right* of the variable names and indicated in color, with higher values indicated in *red*, and lower value indicated in *blue*. These parameter values are plotted with respect to their corresponding SHAP value on the x-axis, where a lower SHAP value indicates that the parameter value influenced the model to predict that the wound would not heal within 4 weeks from the start of treatment, and a higher SHAP value indicates that the parameter value influenced the model to predict that the wound would heal within 4 weeks from the start of treatment. SHAP, Shapley Additive Explanations.

**Figure 3.**
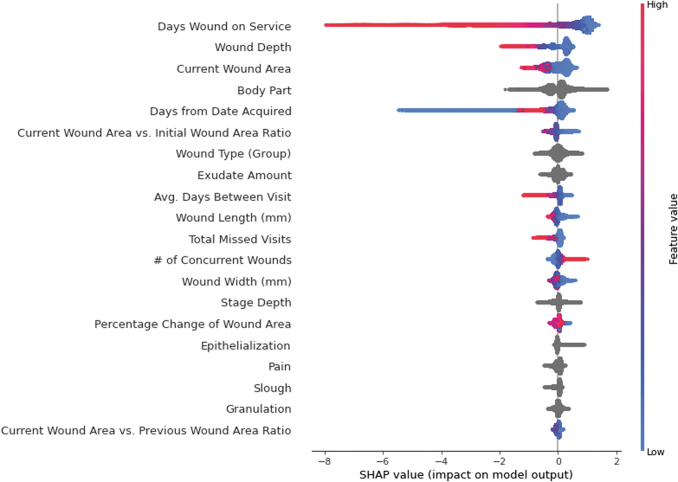
SHAP summary plot for the top 20 most influential variables in the 8-week GBDT predictive model. Variables are listed in descending order of overall influence in the model on the *left-hand side*. Parameter values for variables of individual observations are plotted to the *right* of the variable names and indicated in color, with higher values indicated in *red*, and lower value indicated in *blue*. These parameter values are plotted with respect to their corresponding SHAP value on the x-axis, where a lower SHAP value indicates that the parameter value influenced the model to predict that the wound would not heal within 8 weeks from the start of treatment, and a higher SHAP value indicates that the parameter value influenced the model to predict that the wound would heal within 8 weeks from the start of treatment.

**Figure 4.**
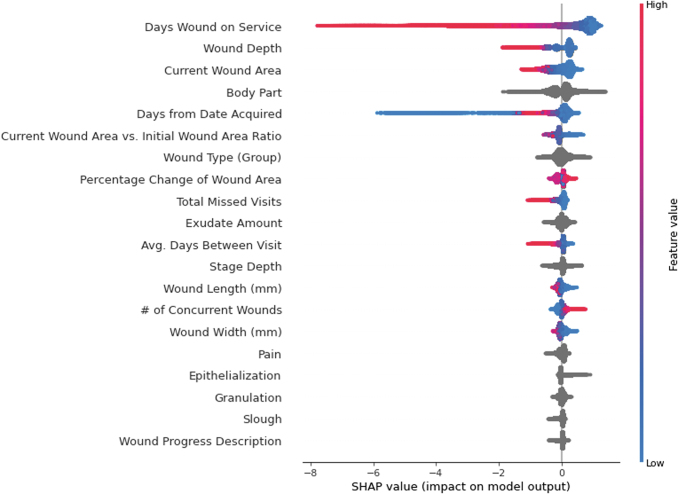
SHAP summary plot for the top 20 most influential variables in the 12-week GBDT predictive model. Variables are listed in descending order of overall influence in the model on the *left-hand side*. Parameter values for variables of individual observations are plotted to the *right* of the variable names and indicated in color, with higher values indicated in *red*, and lower value indicated in *blue*. These parameter values are plotted with respect to their corresponding SHAP value on the x-axis, where a lower SHAP value indicates that the parameter value influenced the model to predict that the wound would not heal within 12 weeks from the start of treatment, and a higher SHAP value indicates that the parameter value influenced the model to predict that the wound would heal within 12 weeks from the start of treatment.

Figures 2–4 show SHAP value summary plots on the testing sets for the 4-, 8-, and 12-week models, respectively. Each plot shows the top 20 most influential variables according to SHAP. The variables are arranged from top to bottom in descending order by magnitude of influence, with the first variable being the most influential. Parameter values for the variables of each observation are represented to the right of the variables' names in color, with higher parameter values for the variables indicated in red and lower values indicated in blue. Parameter values for categorical variables and null continuous variables are represented in gray. Last, the points are plotted with respect to their SHAP value indicated on the horizontal axis at the bottom of the figure. A higher SHAP value indicates the variable influenced the model to predict a higher probability that the wound would heal in the target timeframe. A lower SHAP value indicates the opposite.

In all three models, the number of days the wound had been treated at the time of the visit (“Days Wound on Service”), wound depth (“Depth”), and current wound surface area (“Current Wound Area”) were the top three most influential variables.

For “Days Wound on Service,” a lower SHAP value, indicated in blue, generally influenced the models to predict that the wound would heal before the target timeframe. A higher parameter value of “Days Wound on Service,” indicated in red, influenced the model to predict that the wound would not heal within the timeframe.

For “Depth,” a lower parameter value, indicated in blue, generally influenced the models to predict that the wound would heal before the timeframe of interest. A higher value for “Depth,” indicated in red, had the opposite effect and influenced the models to predict that the wound would not heal before the target timeframe.

For “Current Wound Area,” a lower parameter value, indicated in blue, influenced the models to predict that the wound would heal before the predicted timeframe of interest. A higher value for “Current Wound Area,” indicated in red, influenced the models to predict that the wound would not heal before the respective timeframe.

**Figure 5.**
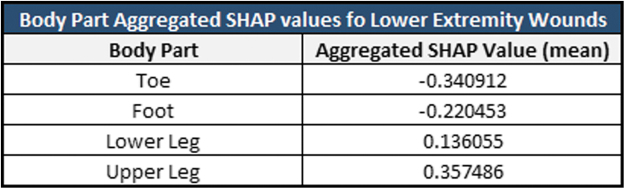
Twelve-weeks target timeframe GBDT model aggregated SHAP values for lower extremity body part locations. A lower SHAP value indicates that the body part location influenced the model to predict the wound would not heal within 12 weeks from the start of treatment, and a higher SHAP value indicates that the body part influenced the model to predict that the wound would heal within 12 weeks from the start of treatment.

Body part location (“Body Part”) was the fourth most influential variable in the models. While this variable is influential, it is not as intuitive to understand visually since it is an integer-encoded nominal categorical variable. To granularly understand how body part location affects predictions, it is necessary to find the aggregated mean SHAP value for “Body Part” to analyze how certain wound locations globally influenced predictions. Figure 5 shows the aggregated average 12-week model SHAP values for lower extremity wounds. “Toe” had the lowest average SHAP value across all wounds, (−0.34), followed by “Foot” (−0.22). “Lower Leg” and “Upper Leg” had slightly higher SHAP values of 0.13 and 0.36. This means that, on average, the model tended to predict that the wounds located on a patient's toe or foot had lower probabilities of healing within the 12-week timeframe than wounds that were on a patient's lower or upper leg.

## DISCUSSION

We developed machine learning models that can accurately predict on each visit whether a chronic wound would heal within 4, 8, and 12 weeks from the start of treatment. These models use clinical features that were curated from structured EHR data and can be deployed in real-time to identify patients with chronic wounds at high risk of not healing within specified timeframes.

To the best of our knowledge, there are no other predictive tools that have demonstrated this accuracy level on every treatment visit for any wound type on a population of this size. Other models developed to predict chronic wound healing time used relatively smaller data sets, had lower accuracy, were limited to specific wound types or healing times, or only made predictions at unique points in time. In contrast, the models in this study were developed on larger data sets that provide predictions for any wound type or visits for multiple timeframes. This makes the algorithms flexible in their ability to accurately generalize across a diverse population.

**Figure 6.**
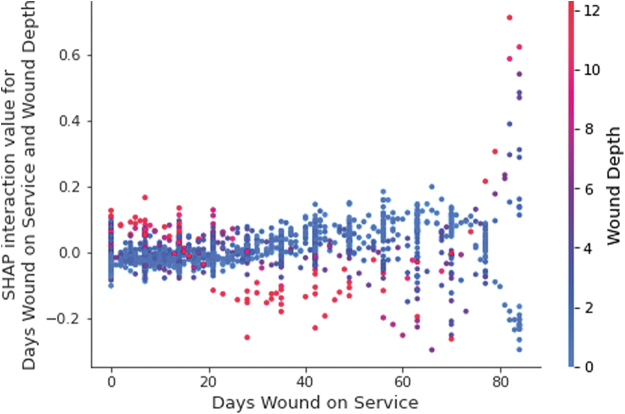
A twelve-week target timeframe GBDT SHAP interaction plot between the independent variables “Days Wound on Service” and “Depth.” Actual parameter values for “Depth” are indicated by color, while actual parameter values for “Days Wound on Service” are indicated on the x-axis. SHAP interaction values are represented on the y-axis on the *left-hand side* of the figure. A positive SHAP value indicates that the observation was influenced to predict that the wound would heal within 12 weeks from the start of treatment. A lower SHAP value indicates that the observation was influenced to predict that the wound would not heal within 12 weeks from the start of treatment. As wound depth is shallower (indicated in *blue*), and “Days Wound on Service” increases, wounds are more likely to heal within 12 weeks. When wounds are of greater depth, indicated in *red*, and patients are further along in treatment, they are less likely to heal in 12 weeks from the start of treatment.

Our models use days in treatment as an independent variable because the design of the study was to make highly accurate predictions on every treatment visit. While days in treatment is highly predictive, the interaction of time and other variables provides for an even more accurate prediction because it can capture more of the longitudinal nature of a patient's progression or degression over the course of treatment. Figure 6 represents a SHAP interaction plot between “Days Wound on Service” and “Depth” for the 12-week GBDT model. In this plot, the parameter values for “Days Wound on Service” are represented on the x-axis, while the parameter values for “Depth” are represented in color. The SHAP interaction values are presented on the y-axis on the left-hand side of the figure. This figure demonstrates that a shallow wound depth, indicated in blue, coupled with a lower “Days Wound on Service” value influences the model to predict that the wound would heal within the 12-week timeframe. As “Days Wound on Service” increases and “Depth” remains shallow, the model is also influenced to predict the wound will heal within the 12-week timeframe. However, as “Days Wound on Service” increases and “Depth” maintains a higher parameter value, the SHAP value decreases, indicating that the model is influenced to predict that the wound will not heal in 12 weeks.

**Figure 7.**
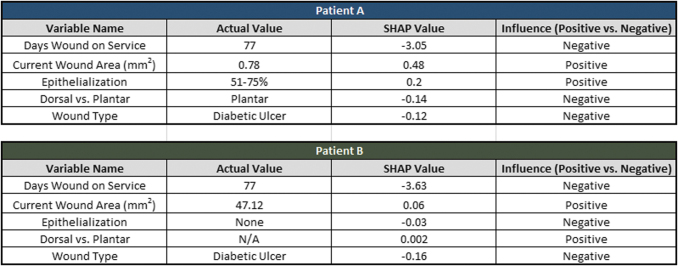
A twelve-week model SHAP value comparison for two patients, A and B. SHAP values are compared for five different covariates: “Days Wound on Service,” “Current Wound Area (mm^2^),” “Epithelialization,” “Dorsal vs. Plantar,” and “Wound Type.” Both patients had diabetic ulcers that had been treated for 77 days. The differing SHAP values for the same variables show how SHAP can unveil observation-specific information about how variables affected single predictions.

The interaction between covariates captured by SHAP is further demonstrated in Fig. 7, which shows five variables and their corresponding 12-week SHAP values for two patients with diabetic foot ulcers. Both patients were 60 years old and had been treated for 77 days. Patient A's wound healed in 12 weeks, while Patient B's did not. The model predicted that Patient A's wound had a 33% chance of healing in the 12-week timeframe, and Patient B had a 3% chance.

Although both patients had the same type of wound and had been treated for the same amount of time, it is evident that other variables in the prediction influenced the way “Days Wound on Service” and “Wound Type” affected both patients differently based on their SHAP values for these variables. For example, “Days Wound on Service” for Patient A had a SHAP value of −3.05, but Patient B had a SHAP value of −3.63. While this variable impacted both patients' predictions negatively, it had more of an effect on Patient B than Patient A. Additionally, both patients had different values for “Epithelialization,” where Patient A had a value of “51–75%,” but Patient B had no value recorded. Patient A received a positive SHAP value of 0.2 for “Epithelialization,” where Patient B had a negative SHAP value of −0.03. This example demonstrates how SHAP can unveil patient-specific information about how a variable influenced a prediction, and the magnitude of the influence.

The analysis has some limitations. First, due to the retrospective nature of the study, no predictions were derived and validated prospectively. Second, no clinical interventions were used as covariates in the models, limiting the prediction to consider patient characteristics, wound characteristics, and time-related variables on each visit. The authors of this article are currently working on incorporating treatments into this analysis. Third, due to the nature of the data, we are unable to capture the nature of recurring wounds. Last, while the analysis was performed on a population of patients from hundreds of facilities across the United States that used a specific EHR, the approach does not harmonize data from other facilities or EHR's. This may limit the models' abilities to perform well on data outside of the facilities from which they were sourced with reliable accuracy and would need further validation on data sets that are not derived from Net Health Systems, Inc.'s Wound Care Analysis Data Set.

In conclusion, this study demonstrates that machine learning algorithms can offer accurate and insightful predictions of chronic wounds at risk of not healing within specific time periods. If integrated thoughtfully within a wound care workflow, clinicians can use these predictions in practice to help earlier identification of patients that are at high risk of having their wounds not heal in 4, 8, and 12 weeks from the start of treatment. This can provide clinicians with important information to facilitate data-driven decision making and may improve patient outcomes and reduce costs associated with non or slow-healing wounds that may prolong care.

## INNOVATION

This study demonstrates that machine learning models can accurately predict chronic wound healing time using historical clinical factors from real-world data curated from EHR's. These models can help clinicians identify patients at risk of non- or slow-healing wounds earlier. SHAP values for predictions can also offer clinicians insight into which variables had a positive or negative affect on a prediction, and the magnitude of the influence with respect to the rest of the covariates. When accurate predictions are used in conjunction with SHAP values, it can drive data-driven decision making that may lead to improved patient care and outcomes.

**KEY FINDINGS**

- The probability of chronic wounds healing within 4, 8, or 12 weeks from the start of treatment can be predicted accurately with EHR data on each treatment visit.
- SHAP can be used to derive global and local importance of covariates in the predictions of chronic wound healing time.
- How long the patient had been in treatment, wound surface area, wound depth, and body part location of wounds were the most influential variables in these models.

## ACKNOWLEDGMENTS AND FUNDING SOURCES

This study did not receive any specific grant from funding agencies in the public, commercial, or not-for-profit sectors. The authors performed this work as part of their regular duties.

## AUTHOR DISCLOSURE AND GHOSTWRITING

The authors state no competing financial interests. The content of this article was expressly written by the authors listed. No ghostwriters were used to write this article.

## ABOUT THE AUTHORS

**Matthew Berezo, MA,** is a Data Scientist for Net Health Systems, Inc. **Joshua Budman, MSE,** is the Vice President of Analytics at Net Health Systems, Inc. **Cathy Thomas Hess, BSN, RN, CWCN,** is the Vice President and Chief Clinical Officer of Wound Care for Net Health Systems, Inc. **Kyle Smith, BS,** is the Vice President of Data Platform for Net Health Systems, Inc. **Daniel Deutscher, PT, PhD,** is a Senior Research Scientist at Net Health Systems, Inc. and the Director of patient-reported outcome measures at the MaccabiTech Institute for Research and Innovation, Maccabi Healthcare Services, in Tel Aviv, Israel. **Deanna Hayes, PT, DPT, MS,** is the Director of Clinical Outcomes and Research for Net Health.

## SUPPLEMENTARY MATERIAL

Supplementary Data S1

Supplementary Data S2

## Supplementary Material

Supplemental data

Supplemental data

## Abbreviations and Acronyms

AUC: area under the ROC curve
DNN: deep feedforward neural network
GBDT: gradient-boosted decision trees
EHR: electronic health records
NPV: negative predictive value
PPV: positive predictive value
ROC: receiver operating characteristic
SD: standard deviation
SHAP: Shapley Additive Explanations
