# Supplementary material for: Predicting Chronic Wound Healing Time Using Machine Learning
Source: Adv Wound Care (New Rochelle). 2022 Mar 24;11(6):281–96. doi: 10.1089/wound.2021.0073 (PMC8982125; doi:10.1089/wound.2021.0073)
Supplement: Supplemental data [file Supp_DataS1.docx]

Supplementary Data S1: Independent Variable Names and Descriptions

Field Name and Description

| AFIB_HST: Comorbidity: patient history of atrial fibrilation |
| --- |
| AGEATVISIT: Patient age (years) |
| ANEMIA_HST: Comorbidity: Patien history of anemia |
| ANTERIOR_POSTERIOR_ID: Wound locatin: anterior vs. posterior |
| ANTIBIOTIC_HST: Comorbidity: Patient history of using antibiotics |
| ANTICOAG_HST: Comorbidity: Patient history of using anticoagulants |
| ARRHYTHMIA_HST: Comorbidity: Patient history of arrhythmia |
| ARTH_HST: Comorbidity: Patient history of arthritis |
| ASTHMA_HST: Comorbidity: Patient history of asthma |
| ATHEROSCLHEARTDIS_HST: Comorbidity: Patient history of Atherosclerosis |
| ATROPHIEBLANCHE_ID: Current wound visit description of atrophied blanche  AVG_DAYS_BETWEEN_VISIT: Average days between visits |
| BLOODDYSCRASIA_HST: Comorbidity: Patient history of Blood dyscrasia |
| BLOODGLUCOSE: Current visit blood glucose measurement |
| BODYPART_ID: Body part location of wound |
| BPH_HST: Comorbidity: Patient history of Benign prostatic hyperplasia |
| BRAWNYINDURATION_ID: Current wound visit description of brawny induration |
| BRIGHTREDGRANULATION_ID: Current wound visit description of bright red granulation |
| BUERGERSDIS_HST: Comorbidity: Patient history of Buerger's disease |
| CAD_HST: Comorbidity: Patient history of coronary artery disesase |
| CALLUS_ID: Current wound visit description of callus |
| CANCER_HST: Comorbidity: Patient history of cancer |
| CATARACTS_HST: Comorbidity: Patient has cataracts |
| CHARCOTFOOT_HST: Comorbidity: Patient history of charcot foot |
| CHF_HST: Comorbidity: Patient history of congestive heart failure |
| CHRON_WOUND_HST: Comorbidity: Patient history of chronic wounds |
| COLORNORMAL_ID: Current wound visit description of normal color |
| COPD_HST: Comorbidity: Patient history of chronic obstructive pulmonary disorder |
| CREPITUS_ID: Current wound visit description of crepitus |
| CRESTSYD_HST: Comorbidity: Patient history of crest syndrome |
| CROHNS_HST: Comorbidity: Patient history of Crohn's disease |
| CVA_HST: Comorbidity: Patient history of cerebrovascular accident |
| CYANOSIS_ID: Current wound visit cyanosis description |
| DAYSWOUNDONSERVICE: Total days wound has been treated at time of visit  DAYS_SINCE_DATE_ACQUIRED: Number of days wound was acquired prior to first treatment visit  DAYS_SINCE_LAST_VISIT: Number of days prior to last visit |
| DEMENT_ALZ_HST: Comorbidity: Patient dementia/Alzheimer's |
| DENUDED_ID: Current wound visit description of denuded |
| DEP_ANX_HST: Comorbidity: Patient history of depression/anxiety  DEPTH: Wound depth  DEPTH_COMP: Current wound depth divided by previous wound depth when wound visit > 1  DEPTH_DIFF: Current wound depth minus previous wound depth when wound visit > 1 |
| DERMATITIS_HST: Comorbidity: Patient history of dermatitis |
| DERMATOMYOCITIS_HST: Comorbidity: Patient history of Dermatomyositis |
| DERMATOPHYTOSIS_HST: Comorbidity: Patient history of Dermatophytosis |
| DIAB_II_HST: Comorbidity: Patient history of Diabetes Type II |
| DIAB_NEURO_HST: Comorbidity: Patient history of Diabetic neuropathy |
| DORSAL_PLANTAR_ID: Dorsal vs. plantar location of wound |
| DRYSCALY_ID: Current wound visit description of dry/scaly |
| DVT_HST: Comorbidity: Patient history of deep vein thrombosis |
| DYSPHAGIA_HST: Comorbidity: patient history of dysphagia |
| ECCHYMOSIS_ID: Current wound visit description of ecchymosis |
| EDEMA_ID: Current wound visit description of edema |
| ENDOCRINE_HST: Comorbidity: Patient history of endocrine |
| EPITHELIALIZATION_F_ID: Current wound visit description of Epithelialization |
| ERYTHEMA_ID: Current wound visit description of erythema |
| ESCHAR_F_ID: Current wound visit description of eschar |
| ESRD_HST: Comorbidity: Patient history of end stage renal disease |
| EXCORIATION_ID: Current wound visit description of excoriation |
| EXUDATEAMOUNT_ID: Current wound visit description of exudate amount |
| EXUDATETYPE_ID: Current wound visit description of exudate type |
| FLUCTUANCE_ID: Current wound visit description of fluctuance |
| FRIABLE_ID: Current wound visit description of friable |
| GENDER_ID: Patient gender |
| GERD_HST: Comorbidity: Patient history of GERD |
| GLAUCOMA_HST: Comorbidity: Patient history of glaucoma |
| GOUT_HST: Comorbidity: Patient history of Gout |
| GRANULATION_F_ID: Current wound visit granulation description |
| HEMIPLEGIA_HST: Comorbidity: Patient hemiplegia history |
| HEMOSIDERINSTAININ_ID: Current wound visit hemosiderin staining description |
| HIV_AIDS_HST: Comorbidity: Patient history of HIV or AID |
| HTN_HST: Comorbidity: Patient history of hypertension |
| HYPERLIPIDEMIA_HST: Comorbidity: Patient history of hyperlipidemia |
| INCONTINENCE_HST: Comorbidity: Patient history of incontinence |
| INDURATION_ID: Current wound visit description of induration |
| INFERIOR_SUPERIOR_ID: Inferior vs. superior location of wound |
| INFL_VAC_HSTUR_HST: Comorbidity: Patient history of having influenza vaccination |
| JOINTPAIN_HST: Comorbidity: Patient history of joint pain |
| JOINTSTIFFNESS_HST: Comorbidity: Patient history of joint stiffness |
| KIDNEY_DIS_HST: Comorbidity: Patient history of kidney disease |
| LEFT_RIGHT_ID: Left vs. right location of wound |
| LENGTH: Current visit wound length (mm) |
| LOCALPULSE_ID: Current wound visit local pulse description |
| LUPUS_HST: Comorbidity: Patient history of lupus |
| LYMPHEDEMA_HST: Comorbidity: patient history of lymphedema |
| MALNUTRITION_HST: Comorbidity: Patient history of malnutrition |
| MEDIAL_LATERAL_ID: Medial vs. lateral location of wound |
| MI_HST: Comorbidity: Patient history of myocardial infarction |
| MOISTURENORMAL_ID: Current wound visit moisture normal description |
| MOIST_ID: Current wound visit moist description |
| MS_HST: Comorbidity: Patient history of multiple sclerosis |
| NEUROPATHY_HST: Comorbidity: Patient history of neuropathy  N_CONCURRENT_WOUNDS: Number of active concurrent wounds |
| OSTEOMYE_HST: Comorbitity: Patient history of osteomyelitis |
| OSTEOPOR_HST: Comorbidity: Patient history of osteoporosis |
| PAIN_ID: Current wound visit pain level |
| PALEGREYGRANULATION_ID: Current wound visit pale/grey granulation description |
| PALLIATIVE_FLG: Indicator if wound is palliative |
| PALLOR_ID: Current wound visit pallor description |
| PARAPLEGIA_HST: Comorbodity: Patient paraplegia |
| PERIPHARTDIS_HST: Comorbidity: Patient history of Peripheral Arterial Disease |
| PERIPH_VASC_HST: Comorbidity: Patient history of Peripheral vascular disease |
| PINKGRANULATION_ID: Current wound visit pink granulation description |
| PNEUMON_HST: Comorbidity: Patient history of pneumonia |
| PNEUMOTHORAXHISTORY_HST: Comorbidity: Patient history of Pneumothorax |
| POLYCYTHEMIAVERA_HST: Comorbidity: Patient history of Polycythemia vera |
| PREV_BLOOD_GLUCOSE: Previous visit blood glucose levels |
| PREV_WOUND_HST: Comorbidity: Patient history of previous wounds |
| PROXIMAL_DISTAL_ID: Proximal vs. distal location of wound |
| PSYCHDISORDER_HST: Comorbidity: Patient history of psychological disorder |
| PV_ATROPHIEBLANCHE_ID: Previous wound visit description of atrophied blanche |
| PV_BRAWNYINDURATION_ID: Previous wound visit description of brawny induration |
| PV_BRIGHTREDGRANULATION_ID: Previous wound visit description of bright red granulation |
| PV_CALLUS_ID: Previous wound visit description of callus |
| PV_COLORNORMAL_ID: Previous wound visit description of normal color |
| PV_CREPITUS_ID: Previous wound visit description of crepitus |
| PV_CYANOSIS_ID: Previous wound visit description of cyanosis |
| PV_DENUDED_ID: Previous wound visit description of denuded  PV_DEPTH: Wound depth from previous visit |
| PV_DRYSCALY_ID: Previous wound visit description of dry/scaly |
| PV_ECCHYMOSIS_ID: Previous wound visit description of ecchymosis |
| PV_EDEMA_ID: Previous wound visit description of edema |
| PV_EPITHELIASLIZATION_F_ID: Previous wound visit description of Epithelialization |
| PV_ERYTHEMA_ID: Previous wound visit description of erythema |
| PV_ESCHAR_F_ID: Previous wound visit description of eschar |
| PV_EXCORIATION_ID: Previous wound visit description of excoriation |
| PV_EXUDATEAMOUNT_ID: Previous wound visit description of exudate amount |
| PV_EXUDATETYPE_ID: Previous wound visit description of exudate type |
| PV_FLUCTUANCE_ID: Previous wound visit description of fluctuance |
| PV_FRIABLE_ID: Previous wound visit description of friable |
| PV_GRANULATION_F_ID: Previous wound visit granulation description |
| PV_HEMOSIDERINSTAININ_ID: Previous wound visit hemosiderin staining description |
| PV_INDURATION_ID: Previous wound visit description of induration |
| PV_LENGTH: Previous visit wound length (mm) |
| PV_LOCALPULSE_ID: Previous wound visit local pulse description |
| PV_MOISTURENORMAL_ID: Previous wound visit moisture normal description |
| PV_MOIST_ID: Previous wound visit moist description |
| PV_PAIN_ID: Previous wound visit pain level |
| PV_PALEGREYGRANULATION_ID: Previous visit pale/grey granulation description |
| PV_PALLOR_ID: Previous wound visit pallor description |
| PV_PINKGRANULATION_ID: Previous wound visit pink granulation description |
| PV_RASH_ID: Previous wound visit rash description |
| PV_RUBOR_ID: Previous wound visit rubor description |
| PV_SCARTISSUE_ID: Previous wound visit scar tissue description |
| PV_SHINY_ID: Previous wound visit shiny description |
| PV_SLOUGH_F_ID: Previous wound visit slough description |
| PV_SSYES_NO_ID: Previous wound visit infection indicator |
| PV_STAGE_DEPTH_ID: Previous wound visit stage depth description |
| PV_TEMPERATURE_ID: Previous wound visit temperature description |
| PV_TEXTURENORMAL_ID: Previous wound visit texture normal description |
| PV_WIDTH: Previous wound visit width (mm) |
| PV_WOUND_PROGRESS_ID: Previous visit wound progress description |
| RACE_ID: Patient race |
| RASH_ID: Current wound visit rash description |
| REYNAUDS_HST: Comorbidity: Patient history of Reynaud's disease |
| RH_ARTH_HST: Comorbidity: Patient history of Rheumatoid arthritis |
| RUBOR_ID: Current wound visit rubor description |
| SCARTISSUE_ID: Current wound visit scar tissue description |
| SEIZURE_HST: Comorbidity: Patient history of seizures |
| SHINY_ID: Current wound visit shiny description |
| SLEEP_AP_HST: Comorbidity: Patient history of sleep apnea |
| SLOUGH_F_ID: Current wound visit slough description |
| SPINABIFIDA_HST: Comorbidity: Patient history of spina bifida |
| SSYES_NO_ID: Current wound visit infection indicator |
| STAGE_DEPTH_ID: Current wound visit stage depth description |
| STROKE_HST: Comorbidity: Patient history of strokes |
| TEMPERATURE_ID: Current wound visit temperature description |
| TET_TOX_VAC_CUR_HST: Comorbidity: Patient history of having tetanus toxoid vaccine |
| TEXTURENORMAL_ID: Current wound visit texture normal description |
| THYROID_DIS_HST: Comorbidity: Patient history of having thyroid disorder |
| TOBACCO_HST: Patient history of tobacco use |
| TOP_TREAT_HST: Comorbidity: Patient history of Topical Treatments  TOTAL_MISSED_VISITS: Total missed visits prior to visit date |
| TYPE_I_DIAB_HST: Comorbidity: Patient history of Type I diabetes |
| VENOUS_HST: Comorbidity: Patient history of venous insufficiency |
| WEAK_MUSC_HST: Comorbidity: Patient history of weak muscles |
| WEIGHT: Patient weight (lb) |
| WIDTH: Current wound visit width (mm) |
| WND_AREA_P_CHANGE: Wound area percentage change from previous vist to current visit |
| WND_VIZ_IDX: Current wound visit number |
| WOUNDPROGRESS_ID: Current wound visit progress description |
| WOUNDTYPE_ID: Wound type |
| WOUND_AREA_CUR: Current wound visit area (mm2) |
| WOUND_AREA_DIFF: Difference in wound area from previous to current visit (mm2)  WOUND_AREA_INITIAL_VS_CURRENT_RATIO : Current wound area divided by initial wound area  WOUND_AREA_CHANGE_RATIO: Current wound area divided by previous wound area when visit number is > 1 |
| WOUND_AREA_PV: Previous visit wound area (mm2) |
| WOUND_AREA_TP_CHANGE: Wound area total percentage change from initial visit to current visit |
