# Supplementary material for: Predicting Chronic Wound Healing Time Using Machine Learning
Source: Adv Wound Care (New Rochelle). 2022 Mar 24;11(6):281–96. doi: 10.1089/wound.2021.0073 (PMC8982125; doi:10.1089/wound.2021.0073)
Supplement: Supplemental data [file Supp_DataS2.docx]

Supplementary Data S2: Random Forest and LightGBM Gradient Boosted Decision Tree Hyperparameter Description and Explored Search Spaces

Random Forest Hyperparameter Descriptions:

Maximum Depth: How deep (n levels) a tree can grow

Minimum samples per split: Minimum number of samples required in a split node for random forest to make the split

Minimum samples per leaf: Minimum number of samples required in a leaf for a random forest to make a split

Maximum features: Random proportion of independent variables to be drawn and considered for a tree in the random forest during model training

LightGBM GBDT Hyperparameter Description:

Evaluation Metric: Loss function evaluated throughout epochs of training that define model success

Maximum depth: How deep (n levels) a tree can grow

Early stopping rounds: Number of rounds without improvement the model can go before it stops training

Column sample by tree: Porportion of random subset of independent variables that can be considered when building a tree in a round of training

Number of leaves: Number of leaf-wise splits a tree can make

Lambda L1: L1 regularization

Lambda L2: L2 regularization

Learning rate: Multiplication performed on each learning iteration’s improvement

Maximum bins: Maximum number of bins that feature values will be bucketed in

Categorical Variables: Specification of integer-encoded categorical features

Random Forest Hyperparameter Search Space:

Maximum Depth: 5 – 150

Minimum samples per split: 2 – 25

Minimum samples per leaf: 2 – 25

Maximum Features: 0.5, 0.75, 0.9, 0.95, 1

LightGBM GBDT Hyperparameter Search Space:

Evaluation metric: AUC, Binary Logarithmic Loss

Maximum depth: 3 – 21

Early stopping rounds: 5 – 100

Column sample by tree: 0.5, 0.75, 0.9, 0.95, 1

Number of leaves: 51 – 256

Lambda L1: 100 – 1500

Lambda L2: 0 – 25

Learning Rate: 0.001 – 0.01
